# Repurposing daclatasvir for MASLD Therapy—A promising step forward with challenges ahead

**DOI:** 10.1016/j.jlr.2025.100857

**Published:** 2025-07-05

**Authors:** Carlos Jose Pirola

**Affiliations:** National Scientific and Technical Research Council and Translational Research in Health Center, Systems Biology of Complex Diseases Department, Maimonides University, Buenos Aires, Argentina

**Keywords:** antiviral drugs, PLIN2, MASH, lipophagy, proteasome

## Abstract

The article discusses the potential of repurposing daclatasvir, an FDA-approved anti-HCV drug, for treating metabolic dysfunction-associated steatotic liver disease (MASLD) and its advanced form, metabolic dysfunction-associated steatohepatitis (MASH). The study by Shu et al. identifies daclatasvir as a potent inhibitor of perilipin-2 (PLIN2), a protein central to lipid droplet stability and metabolic homeostasis. Daclatasvir enhances MARCH6-mediated ubiquitination of PLIN2, leading to its degradation, which reduces lipid accumulation, inflammation, and fibrosis—key features of MASLD and MASH. Preclinical models demonstrate its ability to improve lipid metabolism, reduce inflammation, and alleviate liver fibrosis. Despite promising findings, challenges remain. Clinical trials are needed to validate its efficacy and safety in humans, as animal models cannot fully replicate the multifactorial nature of MASLD. Long-term safety and potential off-target effects also require evaluation, especially since PLIN2 may protect against other liver conditions. The study highlights the need for broader screening of FDA-approved drugs and exploration of alternative pathways for PLIN2 regulation. While daclatasvir shows promise, further research is essential to address these gaps and advance its clinical application for MASLD therapy. The findings underscore the potential of drug repurposing as a cost-effective strategy for unmet medical needs.

The global burden of metabolic dysfunction-associated steatotic liver disease (MASLD) and its advanced form, metabolic dysfunction-associated steatohepatitis (MASH), continues to rise with limited therapeutic options available, except for the conditionally approved resmetirom (1) and some promising candidates in the pipeline, such as glucagon-like peptide-1 receptor (GLP1R) agonists (2). Then, leveraging drug repurposing strategies, which reduce development time and costs, offers a promising avenue to address the unmet clinical needs of patients suffering from these metabolic liver diseases (3). In fact, using overrepresentation analysis of MASLD-associated targets and a drug-related functional database (drug_GLAD4U) it could be predicted that several drug categories may target many components of the druggable MASLD genome/proteome, including anti-infective drugs (3).

In this context, the study by Shu *et al.* (4) offers a compelling case for repurposing daclatasvir, a Food and Drug Administration-approved anti-hepatitis C virus (HCV) drug, as a novel therapeutic candidate for MASLD and MASH. However, while the findings are robust and innovative, they also highlight critical gaps that must be addressed before clinical translation.

One of the study’s key strengths lies in its identification of daclatasvir as a potent inhibitor of perilipin-2 (PLIN2), a protein central to lipid droplet stability, metabolic homeostasis, and the lipophagy process. Through a series of well-designed experiments, the authors demonstrate that daclatasvir enhances MARCH6 (membrane-associated ring-CH-type finger 6)-mediated lysine 11 (K11)-type ubiquitination of PLIN2, leading to its degradation. This mechanism effectively reduces lipid accumulation, inflammation, and fibrosis—hallmarks of MASLD and MASH. The specificity of daclatasvir’s action is underscored by its binding to key residues (Ser229 and Glu241) in PLIN2, with mutations at these sites abolishing daclatasvir’s effects.

The study involves a comprehensive experimental approach, employing in vitro cell models, diet-induced mouse models, molecular docking, and isothermal titration calorimetry. These methodologies validate the therapeutic potential of daclatasvir and its interaction with PLIN2. Furthermore, the drug’s ability to improve lipid metabolism, reduce inflammatory responses, and alleviate liver fibrosis in both high-fat high-cholesterol and methionine-choline-deficient diet-induced mouse models of MASH is noteworthy. These findings suggest that daclatasvir could offer broad therapeutic benefits for MASLD patients, addressing both metabolic and inflammatory dysregulation.

Despite these strengths, the study raises several questions that warrant further investigation. First and foremost, the clinical relevance of daclatasvir in MASLD and MASH remains uncertain. Although the preclinical data are promising, the absence of clinical trials leaves a significant gap in understanding the efficacy and safety of this treatment in humans. The transition from animal models to human applications is fraught with challenges, particularly given the multifactorial nature of MASLD, which involves genetic, environmental, and lifestyle factors that are difficult to replicate in diet-induced mouse models. Despite these caveats, there is some evidence that daclatasvir, when used in combination with other anti-HCV drugs (particularly sofosbuvir and daclatasvir or sofosbuvir and ledipasvir), may be effective and safe in ameliorating liver fat infiltration while achieving a sustained virological response after 12 weeks in nondiabetic, chronically infected patients with HCV (5).

In addition, the study’s focus on daclatasvir limits exploration of other potential candidates from the Food and Drug Administration-approved drug library. Although the authors undertook a preliminary screening of the effects of a panel composed of acyclovir, daclatasvir, dasabuvir, famciclovir, glecaprevir, idarubicin, lamivudine, ombitasvir, paritaprevir, ribavirin, sofosbuvir, and tenofovir, as mentioned, other anti-HCV drugs may be equally effective. A broader screening might reveal additional therapeutic options, potentially offering synergistic effects or alternatives with fewer side effects. Referring to side effects, the long-term safety of daclatasvir when repurposed for MASLD remains unaddressed. Originally developed as an antiviral drug, its impact on nonviral liver conditions and potential off-target effects required careful evaluation. This is particularly important, as PLIN2 may be protective against other liver noxious agents, such as hepatitis B virus (6). In addition, the transcriptomic analysis highlights improvements in lipid metabolism and inflammation; however, the study does not delve into specific pathways that may be affected by daclatasvir beyond PLIN2 degradation, but it showed a plethora of downregulated transcripts. In both models, Huh-7 and diet-induced MASH in mice, downregulated transcripts are overrepresented in pathways related to diverse virus infection (Epstein–Barr, measles, influenza A, and human papilloma virus) and other infectious agents (Chagas and Yersinia) indicating potential adverse off-target effects (Fig. 1).

**Fig. 1:**
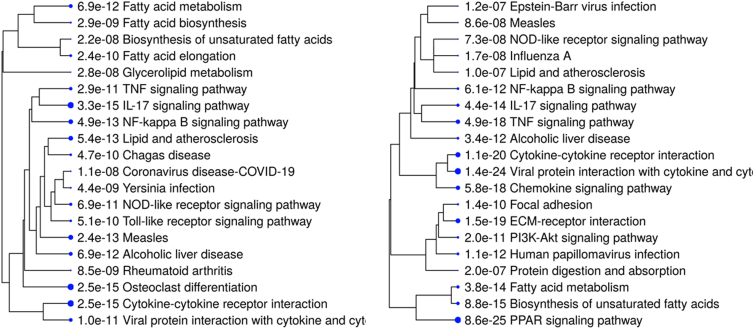
A hierarchical clustering tree summarizes the correlation among significantly enriched pathways. Pathways with many shared genes are clustered together. *Bigger dots* indicate more significant *P*-values. Gene enrichment is analyzed and their pathways from the KEGG pathway database are retrieved by the web-based program ShinyGO 0.82 (https://bioinformatics.sdstate.edu/go/) with a false discovery rate (FDR) of 0.05, and the pathways shown are limited to 20. The *left panel* shows the results of including the list of downregulated transcripts from the experiment in HuH-7 cells after 18 h of treatment with daclatasvir under palmitic acid/oleic acid (0.25 mM/0.5 mM) stimulation (Figure 2 of Shu et al.’s study (4)). The *right panel* shows the results of including the list of downregulated transcripts from the experiment in liver tissues from MCD diet-induced MASH mice following 3 weeks of daclatasvir or vehicle treatment (Figure 3 of Shu et al.’s study (4)). MASH, metabolic dysfunction-associated steatohepatitis; MCD, methionine-choline-deficient; KEGG, Kyoto Encyclopedia of Genes and Genomes.

Mechanistically, while the study provides valuable insights into PLIN2 degradation, the broader implications of this process on lipid metabolism and mitochondrial function remain largely unexplored. The role of other E3 ligases, such as UBR1, is briefly mentioned but not thoroughly investigated leaving room for further research into alternative pathways that may contribute to PLIN2 regulation.

The finding that PLIN2 inhibition improves liver fat infiltration and its consequences is robust. A missense variant of PLIN2 (Ser251Pro) was associated with a trend toward reduced liver fat accumulation in humans, although the variant is not associated with MASLD (at that time, nonalcoholic fatty liver disease (NAFLD)) in several cohorts (7). Furthermore, the human *PLIN2-Pro251* overexpression in *Plin2* KO mice attenuated high-fat, high-fructose, high-cholesterol diet-induced hepatic steatosis, accompanied by less liver oxidative stress, compared with human *PLIN2-Ser251* overexpression (7).

Finally, the study by Shu *et al.* adds another tool to the armamentarium, confirming that decreasing PLIN2 is an effective way to improve MASLD. Exercise (8), diet interventions, and GLP1R agonists (9) improve MASLD, at least in part, by decreasing *Plin2* expression in rodent models of MASLD. Daclatasvir seems to be more appropriate than knocking down *Plin2* with siRNA. GalNAc-siPlin2 significantly improved hepatic steatosis, inflammation, and fibrosis in high-fat/high fructose-induced MASH models compared to the control group (10). However, beyond the proof of concept, siRNAs are difficult to administer and are likely to be significantly more expensive.

In conclusion, Shu *et al.* presented a groundbreaking study that highlights the potential of repurposing daclatasvir for the treatment of MASLD. The findings are a significant step forward in addressing the limitations of current treatments, offering hope for more effective and accessible therapeutic options. Generally, it shows a way to explore drug repurposing for MASLD treatment further. However, the road to clinical application is long and requires addressing key gaps, including clinical validation, safety profiling, and broader exploration of drug candidates and mechanisms. As the scientific community continues to seek solutions for MASLD and MASH, this study serves as both an inspiration and a call to action for further research and innovation.

## Conflict of Interest

The author declares that he has no conflict of interest with the contents of this article.

## References

[1] Sookoian S., Pirola C.J. (2024). Resmetirom for treatment of MASH. Cell.

[2] Tacke F., Horn P., Wong V.W.-S., Ratziu V., Bugianesi E., Francque S. (2024). EASL–EASD–EASO Clinical Practice Guidelines on the management of metabolic dysfunction-associated steatotic liver disease (MASLD). J. Hepatol..

[3] Sookoian S., Pirola C.J. (2020). Precision medicine in nonalcoholic fatty liver disease: New therapeutic insights from genetics and systems biology. Clin. Mol. Hepatol..

[4] Shu R., Tian S., Qu W., Yang J., Shi W., Li X. (2025). Hepatoprotective drug screening identifies daclatasvir a promising therapeutic candidate for MASLD by targeting PLIN2. J. Lipid Res..

[5] El-Ghandour A., Youssif T., Ibrahim W., Abdelsattar H.A., Bawady S.A.E., Wagih M. (2023). The effect of different direct antivirals on hepatic steatosis in nondiabetic and naïve hepatitis C-infected Egyptian patients. Egypt J. Intern. Med..

[6] Delpino M.V., Quarleri J. (2024). Perilipin 2 inhibits replication of hepatitis B virus deoxyribonucleic acid by regulating autophagy under high-fat conditions. World J. Virol..

[7] Scorletti E., Saiman Y., Jeon S., Schneider C.V., Buyco D.G., Lin C. (2024). A missense variant in human perilipin 2 (PLIN2 Ser251Pro) reduces hepatic steatosis in mice. JHEPReport.

[8] Fang C., Liu S., Yang W., Zheng G., Zhou F., Gao X. (2024). Exercise ameliorates lipid droplet metabolism disorder by the PLIN2–LIPA axis-mediated lipophagy in mouse model of non-alcoholic fatty liver disease. Biochim. Biophys. Acta Mol. Basis Dis..

[9] Pontes-da-Silva R.M., de Souza Marinho T., de Macedo Cardoso L.E., Mandarim-de-Lacerda C.A., Aguila M.B. (2022). Obese mice weight loss role on nonalcoholic fatty liver disease and endoplasmic reticulum stress treated by a GLP-1 receptor agonist. Int. J. Obes..

[10] Wang Y., Zhou J., Yang Q., Li X., Qiu Y., Zhang Y. (2024). Therapeutic siRNA targeting PLIN2 ameliorates steatosis, inflammation, and fibrosis in steatotic liver disease models. J. Lipid Res..

